# Testosterone replacement therapy and the risk of adverse cardiovascular outcomes and mortality

**DOI:** 10.1186/s12610-019-0085-7

**Published:** 2019-03-29

**Authors:** Kevin M. Pantalone, Joyce George, Xinge Ji, Michael W. Kattan, Alex Milinovich, Janine M. Bauman, Bartolome Burguera, Robert S. Zimmerman, Anita D. Misra-Hebert

**Affiliations:** 1 Department of Endocrinology, Cleveland Clinic, Endocrinology and Metabolism Institute, 9500 Euclid Avenue, Desk F-20, Cleveland, Ohio 44195 USA; 2 Southeast Georgia Health System, 2500 Starling St Suite 501, Brunswick, GA 31520 USA; 3 Quantitative Health Sciences, Cleveland Clinic, Desk JJN3, Cleveland, Ohio 44195 USA; 4 Medicine Institute, Cleveland Clinic, Desk G-10, Cleveland, Ohio 44195 USA

**Keywords:** Testosterone replacement therapy, Male hypogonadism, Cardiovascular risk, Mortality

## Abstract

**Background:**

The risk of adverse cardiovascular events and mortality associated with testosterone replacement therapy is controversial. The purpose of this report was to evaluate the effect of testosterone replacement therapy (TRT) in men with secondary hypogonadism on the risk of myocardial infarction (MI), stroke (CVA) or all-cause mortality.

**Methods:**

A retrospective cohort study was conducted using the Cleveland Clinic’s electronic health record. Men ≥40 years of age, with at least two testosterone levels < 220 ng/dL, with one level obtained between 7 am and 10 am, were identified. Men with primary hypogonadism, secondary hypogonadism related to overt hypothalamic pituitary pathology, human immunodeficiency virus infection, metastatic cancer, and select contraindications to TRT, were excluded. Men exposed to TRT were matched to controls that were not exposed. A survival analysis was performed on the composite outcome of MI, CVA, or all-cause mortality.

**Results:**

One hundred sixty-five patients exposed to TRT (treatment group) were matched with 210 not exposed to TRT (comparison group). The prevalence of established cardiovascular disease (CVD) was 20.0% in the treatment group vs. 17.1% in the comparison group (*P* = 0.478). The median [interquartile range (IQR)] age (years) and BMI (kg/m^2^) were 55 (49, 62) and 35.6 (32.1, 40.1) in the treatment group, and 55 (49, 61.7) and 36.3 (32.1, 40.8) in the comparison group, respectively. There were 12 (7.3%) events observed in the treatment group, and 16 (7.6%) in the comparison group. The median time (years) to the composite event was 2.1 (IQR 0.9, 4.6) and 1.8 (IQR 0.6, 3.4) for treatment and comparison groups, respectively. No difference in the risk of the combined cardiovascular endpoint was observed between the treatment group vs the comparison group, hazard ratio (HR) 0.81 (95% Confidence Interval [CI]: 0.38–1.71; *P* = 0.57).

**Conclusion:**

In hypogonadal men with a modest prevalence of established CVD, TRT was not observed to confer a protective or adverse effect on the risk of MI, CVA or all-cause mortality.

## Résumé

**Contexte:**

Le risque d’événements cardiovasculaires indésirables et de mortalité associé au traitement de substitution de la testostérone est controversé. Le but du présent article est d’évaluer chez les hommes qui présentent un hypogonadisme secondaire l’effet du traitement de substitution de la testostérone (TST) sur le risque d’infarctus du myocarde (IM), d’accident cérébrovasculaire (ACV) ou de mortalité toutes causes confondues.

**Patients et Méthodes:**

Une étude de cohorte rétrospective a été menée en utilisant les dossiers de santé électroniques de la Clinique Cleveland. Ont été identifiés les hommes âgés de plus de 40 ans qui avaient au moins deux dosages de testostérone inférieurs à 220ng/dl, dont l’un obtenu le matin entre 7 et 10 heures. Ont été exclus les hommes qui présentaient un hypogonadisme primaire, un hypogonadisme secondaire lié à une pathologie hypothalamo-hypophysaire évidente, une infection par le virus de l’immunodéficience humaine, un cancer métastatique, et ceux qui présentaient des contrindications déterminées au TST. Les hommes exposés au TST ont été appariés à des témoins non exposés au TST. Une analyse de survie a été réalisée sur le paramètre composite incluant l’IM, l’AVC et la mortalité toutes causes confondues.

**Résultats:**

165 patients exposés au TST (groupe traité) ont été appariés à 210 hommes non exposés au TST (groupe de référence). La prévalence de maladie cardiovasculaire (MCV) établie était de 20.0% dans le groupe traité versus 17.1% dans le groupe de référence (P=0.478). La médiane (écart interquartile (EI)) de l’âge et de l’indice de masse corporelle (IMC) était respectivement de 55 (49, 62) ans et de 35.6 (32.1, 40.1) kg/m2 dans le groupe traité, et respectivement de 55 (49, 61.7) ans et 36.3 (32.1, 40.8) kg/m2 dans le groupe de référence. Il y eut 12 événements indésirables (7.3%) dans le groupe traité, et 16 (7.6%) dans le groupe de référence. La médiane du temps (en années) des événements composites était de 2.1 (EI 0.9, 4.6) et de 1.8 (EI 0.6, 3.4) respectivement pour les groupes traité et de référence. Aucune différence n’a été observée en ce qui concerne le risque cardiovasculaire entre le groupe traité et le groupe de référence, rapport de risque (RR) 0.81 (Intervalle de Confiance à 95% [IC]: 0.38-1.71; *P* = 0.57).

**Conclusion:**

Chez les hommes qui présentent un hypogonadisme et une faible prévalence de maladie cardiovasculaire (MCV) établie, le TST n’apparait pas conférer un effet protecteur ou défavorable sur le risque de d’infarctus du myocarde (IM), d’accident cérébrovasculaire (ACV) ou de mortalité toutes causes confondues.

**Mots-clés:**

Traitement de substitution de la testostérone, Hypogonadisme masculin, Risque cardiovasculaire, Mortalité.

## Introduction

The effect of testosterone replacement therapy (TRT) on cardiovascular risk has been controversial [1, 2]. Concerns regarding the cardiovascular safety of TRT first surfaced in 2010 when Basaria et al. reported that in a population of older men with limitations in mobility and a high prevalence of chronic disease who were randomized to receive placebo gel or testosterone gel, that the application of a testosterone gel was associated with an increased risk of cardiovascular adverse events (23 subjects in the testosterone group, as compared with 5 in the placebo group, experienced cardiovascular-related adverse events) [3]. Since this report, many retrospective studies regarding the safety of testosterone replacement therapy in hypogonadal men have been published with mixed results [4–8].

However, it is important to note that the designs and study populations in these reports differed significantly, which may explain the heterogeneity of the observed results. While there have been many prospective randomized controlled trials published recently regarding the benefits of testosterone replacement therapy on sexual function, mood and depression, bone mineral density, and anemia, the cardiovascular safety and the risk of mortality associated with TRT remains unclear [9–12]. A placebo controlled clinical trial recently reported an increase in coronary artery non-calcified plaque volume in men receiving TRT; however, the clinical implications of this observation have not been determined [13].

What appear to be important clinical parameters to consider when deciding to initiate TRT in hypogonadal men are their clinical history and functional status. It would seem to be important to distinguish TRT in younger males with primary hypogonadism (e.g. Klinefelter Syndrome), or secondary hypogonadism related to overt hypothalamic-pituitary pathology (e.g., pituitary adenoma) vs. initiating TRT in hypogonadal males as a result of aging or chronic conditions like obesity as the risks vs. benefits in these distinct populations with different underlying pathophysiologies are likely to be dissimilar. The older population, and/or those with many comorbid conditions like obesity, generally have a higher likelihood of having established cardiovascular disease (CVD), or numerous risk factors for CVD, and thus, may be more susceptible to potential adverse events that could result from TRT (increased risk of thrombosis, elevated hematocrit (HCT), etc.). The underlying pathophysiology driving the development of secondary hypogonadism should be considered in order to appropriately assess the risks vs. benefits of TRT, particularly in men of advanced age, those with a high-comorbidity burden, and/or established CVD. The objective of this report was to evaluate the effect of testosterone replacement therapy (TRT) on the risk of myocardial infarction (MI), stroke (CVA) or all-cause mortality, in men with secondary hypogonadism unrelated to overt hypothalamic-pituitary pathology managed at our institution.

## Materials and methods

A retrospective cohort study was conducted using the electronic health record (EHR) in a large, integrated healthcare system from 2005 to 2015. Men ≥40 years of age with at least two testosterone levels < 220 ng/dL were identified. To improve the accuracy of the diagnosis of testosterone deficiency, we required one of the two testosterone levels be obtained between 7 am and 10 am, through confirming the time stamp in the EHR lab report. To focus our analysis on men with secondary hypogonadism who would receive testosterone replacement in routine practice, men with International Classification of Diseases, Ninth Revision (ICD-9) codes for primary hypogonadism (257.1, 758.7), secondary hypogonadism related to overt hypothalamic/pituitary pathology (194.3, 227.3, 237.0, 253.X, 255.0, 255.3, 758.X, 962.X, 275.0), or patients with sexual or gender identify disorders who would all be appropriate candidates for testosterone replacement (302.X except 302.7X and 302.9), were excluded. Patients with increased risk for mortality with diagnoses of human immunodeficiency virus (HIV) infection or metastatic cancer and those men with likely contraindications to testosterone treatment including history of prostate cancer, prostate specific antigen > 4 ng/mL, elevated hematocrit (above upper limit of lab reference range), or a history of previous thromboembolic disease were excluded. There were no other medical conditions that were grounds for exclusion.

Men exposed to TRT (treatment group) were matched to hypogonadal men who were not exposed to testosterone (comparison group). Testosterone exposure was defined as documentation of a prescription for an injectable or topical form of TRT within the EHR. Since patients could start TRT at any time point during their interval of low testosterone, patients in the treatment group were initially matched to the comparison group based upon duration of low testosterone exposure in the dataset prior to treatment, and then matched using full matching method on the following variables: age, income, diabetes, smoking status, low-density lipoproteins (LDL) level, hypertension, statin use, body mass index (BMI), prior cardiovascular or cerebrovascular disease at baseline, and Charlson Comorbidity Index score (Charlson score was categorized as Charlson score = 0; Charlson score = 1; Charlson score > 1) [14]. ICD-9 codes were used to define prior/established cardiovascular or cerebrovascular disease (Table 1).

**Table 1:** ICD-9 Codes used to define established cerebrovascular and cardiovascular disease

Cerebrovascular Disease^a^	
430, 430–438.99, 431, 432, 432.0, 432.1, 432.9, 433.0, 433.00, 433.01, 433.1, 433.10, 433.11,	
434.01,434.1434.10,434.11,434.9434.90,434.91,435.0,435.1435.2435.3435.8435.9436,	
437.0,437.1437.2437.3437.4437.5437.6437.7437.8438.0,438.10,438.11,438.12,438.13,	
438.14,438.19,438.2438.20,438.21,438.22.438.3438.30,438.31,438.32,438.4438.40,438.41,	
438.42,438.5438.50,438.51,438.52,438.53,438.6438.7438.81,438.82,438.83,438.84,438.85,438.89,438.9	
Cardiovascular Disease^a^	
411,411.0,411.1411.8411.81,411.89,412,413,413.0,413.1413.9,414,414.0,414.00,414.01,	
414.02,414.03,414.04,414.05,414.06,414.07,414.1414.10,414.11,414.12,414.19,414.2414.3,	
414.4414.8414.9429.2,440,440.9996.03,V45.81,V45.82	

^a^These ICD-9 codes had to be present in the past medical history or problem list fields of the EHR only to be classified as prior/established CVD. They could not be present as an encounter diagnosis (inpatient or outpatient) during the study period

For a patient treated with TRT, we recorded the untreated time period since baseline as X months and restarted the patient clock to 0 at treatment initiation. Then we tried to match this patient to a comparison group patient (no TRT) that had more than X months of follow-up since baseline. For the matched comparison group patient, we restarted the clock to 0 at X months as well.

In order to be classified as prior/established CVD, the ICD-9 codes listed in Table 1 had to be present only in the past medical history or problem list fields of the EHR; they could not be present as an encounter diagnosis (inpatient or outpatient) during the study period. Income was defined as the 2011–2015 five year estimates of median household income at the block group level obtained from the American Community Survey conducted by the United States Census Bureau [15]. Full matching divided the full sample of all treatment and all comparison patients into a series of matched sets, such that each set contained either one treatment individual and multiple comparison individuals or one comparison individual and multiple treatment individuals. The ratio of treatment: comparison individuals in each matched set depended on the relative number of treatment and comparison individuals with the same matching variables. The maximum and minimum ratio were 2:1 and 1:5. By using full matching, we were able to make use of more observations than pair matching. Standardized mean difference (SMD) was used as a balance measure of the individual covariates after matching (Tables 2 and 3).

**Table 2:** Primary analysis, baseline covariates after matching

Matching Variable	Comparison Group (No TRT^a^) *N* = 210	Treatment Group (TRT^a^) *N* = 165	*P*-value	Standardized Mean Difference
Median First Testosterone, ng/dL (IQR)	173 (146, 198)	179 (153, 203)	0.397	0.265
Median Second Testosterone, ng/dL (IQR)	170 (142, 198)	172 (138, 193)	0.798	0.085
Median Income, United States Dollars (IQR)	54,468 (47,300, 69,769)	59,311 (45,124, 70,631)	0.612	0.440
Median Age, years (IQR)	55 (49, 61.7)	55 (49, 62)	0.815	0.033
Diabetes (N, %)	88 (41.9%)	67 (40.6%)	0.800	0.026
Tobacco Use (N, %)	1 (0.5%)	1 (0.6%)	0.864	0.018
LDL > 130 mg/dL	18 (8.6%)	20 (12.2%)	0.258	0.117
Median LDL, mg/dL (IQR)	92 (73, 114)	91 (68, 113)	0.458	0.058
Hypertension (N, %)	142 (67.6%)	111 (67.3%)	0.943	0.007
Statin (N, %)	106 (50.5%)	86 (52.1%)	0.752	0.033
BMI ≥ 30 kg/m^2^	192 (91.9%)	144 (87.3%)	0.191	0.135
Median BMI, kg/m^2^ (IQR)	36.3 (32.1, 40.8)	35.6 (32.1, 40.1)	0.290	0.291
Previous Cardiovascular Disease (N, %)	36 (17.1%)	33 (20.0%)	0.478	0.074
Charlson Comorbidity Index (N, %)			0.532	0.117
0	86 (41%)	61 (37%)		
1	46 (21.9%)	44 (26.7%)		
> 1	78 (37.1%)	60 (36.3%)		
**Composite Outcome (N, %)**	16 (7.6%)	12 (7.3%)		
Myocardial Infarction (N, %)	3 (1.4%)	4 (2.4%)		
Stroke (N, %)	10 (4.8%)	6 (3.6%)		
Death (N, %)	3 (1.4%)	2 (1.2%)		

^a^TRT: Testosterone Replacement Therapy

IQR: Interquartile range

LDL: Low-density lipoproteins

BMI: Body mass index

Patients were matched by age, median income, diabetes, tobacco, hypertension, LDL, stain, aspirin, body mass index (BMI), previous cardiovascular disease (previous acute myocardial infarction or acute cerebrovascular accident, or cerebrovascular or cardiovascular disease), and Charlson Comorbidity Index. Median matched exposure time was 1 month (IQR: 0, 4), max 78 months

A patient treated with TRT with X months of exposure time between baseline and the initiation of treatment was matched to a comparison group patient (no TRT) that had more than X months of follow up since baseline. This period of time (X months) is the matched exposure time

**Table 3:** Primary analysis, baseline covariates after matching, stratified by age

Matching Variable	Age < 65 years *N* = 304	Age ≥ 65 years *N* = 71	*P*-value	Standardized Mean Difference
TRT^a^ (N, %)	132 (43.4%)	33 (46.5%)	0.738	0.061
Median First Testosterone, ng/dL (IQR)	175 (145, 202)	185 (161, 196)	0.291	0.126
Median Second Testosterone, ng/dL (IQR)	169 (139, 195)	176 (141, 195)	0.526	0.040
Median Income, United States Dollars (IQR)	54,440 (44,810, 71,425)	61,069 (45,653, 66,588)	0.538	0.071
Median Age, years (IQR)	54 (48, 59)	69 (66, 74)	< 0.001	0.687
Diabetes (N, %)	116 (38.2%)	39 (54.9%)	0.014	0.341
Tobacco Use (N, %)	2 (0.1%)	0	1	0.115
LDL > 130 mg/dL	37 (12.2%)	1 (1.4%)	0.013	0.438
Median LDL, mg/dL (IQR)	95 (74, 117)	79 (59, 107)	0.001	0.536
Hypertension (N, %)	193 (63.5%)	60 (84.5%)	0.001	0.494
Statin (N, %)	147 (48.4%)	45 (63.4%)	0.032	0.306
BMI ≥ 30 kg/m^2^	276 (90.8%)	60 (84.5%)	0.179	0.192
Median BMI, kg/m^2^(IQR)	36.3 (32.2, 41.0)	34.1 (31.1, 37.9)	0.004	0.323
Previous Cardiovascular Disease (N, %)	35 (11.5%)	34 (47.9%)	< 0.001	0.868
Charlson Comorbidity Index (N, %)			< 0.001	1.068
0	134 (44.1%)	13 (18.3%)		
1	85 (28.0%)	5 (7.0%)		
> 1	85 (28.0%)	53 (74.6%)		
**Composite Outcome (N, %)**	15 (4.9%)	13 (18.3%)		
Myocardial Infarction (N, %)	5 (1.6%)	2 (2.8%)		
Stroke (N, %)	8 (2.6%)	8 (11.3%)		
Death (N, %)	2 (0.7%)	3 (4.2%)		

^a^TRT: Testosterone Replacement Therapy

IQR: Interquartile range

LDL: Low-density lipoproteins

BMI: Body mass index

Patients were matched by age, median income, diabetes, tobacco, hypertension, LDL, stain, aspirin, body mass index (BMI), previous cardiovascular disease (previous acute myocardial infarction or acute cerebrovascular accident, or cerebrovascular or cardiovascular disease), and Charlson Comorbidity Index. Median matched exposure time was 1 month (IQR: 0, 4), max 78 months

A patient treated with TRT with X months of exposure time between baseline and the initiation of treatment was matched to a comparison group patient (no TRT) that had more than X months of follow up since baseline. This period of time (X months) is the matched exposure time

For baseline demographic and clinic characteristics, differences between the treatment and comparison groups were analyzed by using chi-square tests for categorical variables and t-tests for continuous data. A composite post-baseline outcome for low testosterone was defined as the first event of acute MI (ICD-9 codes 410.02, 410.90, 410.70, 410.42, 410.80, 410.71, 410.10, 410.72, 410.50, 410.40, 410.41, 410.60, 410.91, 410.30, 410.00, 410.11, 410.8, 410.82, 410.01, 410.1, 410.21, 410.51, 410.22, 410.81, 410.20, 410.92, 410.31, 410.5, 410.4, 410.12, 410.7, 410.61, 410.32, 410.52, 410.2, 410.0, 410.62, 410.3, 410.6), acute stroke (ICD-9 codes 431, 433.0, 433.00, 433.01, 433.1, 433.10, 433.11, 433.2, 433.20, 433.21, 433.3, 433.30, 433.31, 433.8, 433.80, 433.81, 433.9, 433.90, 433.91, 434.0, 434.00, 434.01, 434.1, 434.10, 434.11, 434.9, 434.90, 434.91, 436), or death (all-cause mortality) defined by vital status within the Cleveland Clinic EHR, Ohio Death Index, or Social Security Death Index (data available through 10/31/2011). Patients were censored at the time of the first composite outcome after baseline/diagnosis of low testosterone. The above ICD-9 codes that defined an acute MI or acute stroke had to occur within an encounter (inpatient or outpatient) to qualify as an acute MI or acute stroke event/diagnosis. Relative risks of TRT were estimated by calculating the hazard ratio by Multivariate Cox proportional hazards regression. A *P*-value < 0.05 was considered statistically significant. All statistical analyses were performed by using R system for statistical computing.

This research was approved by Cleveland Clinic’s Institutional Review Board. A waiver of informed consent was granted.

## Results

The 165 patients exposed to TRT (treatment group) were matched with 210 patients not exposed to TRT (comparison group). The median (Interquartile Range [IQR]) age (years), and the prevalence of established CVD (%), in the treatment group vs. the comparison group were 55 (49, 62) vs. 55 (49, 62), *P* = 0.815 and 20.0% vs. 17.1%, *P* = 0.478, respectively. The majority of patients in both the treatment and comparison groups were obese, 87.3% vs 91.9%, respectively (*P* = 0.19). Please see Table 2 for a complete list of matched variables, and Table 3 for a complete list of matched variables stratified by age < or ≥ 65 years.

**Fig. 1:**
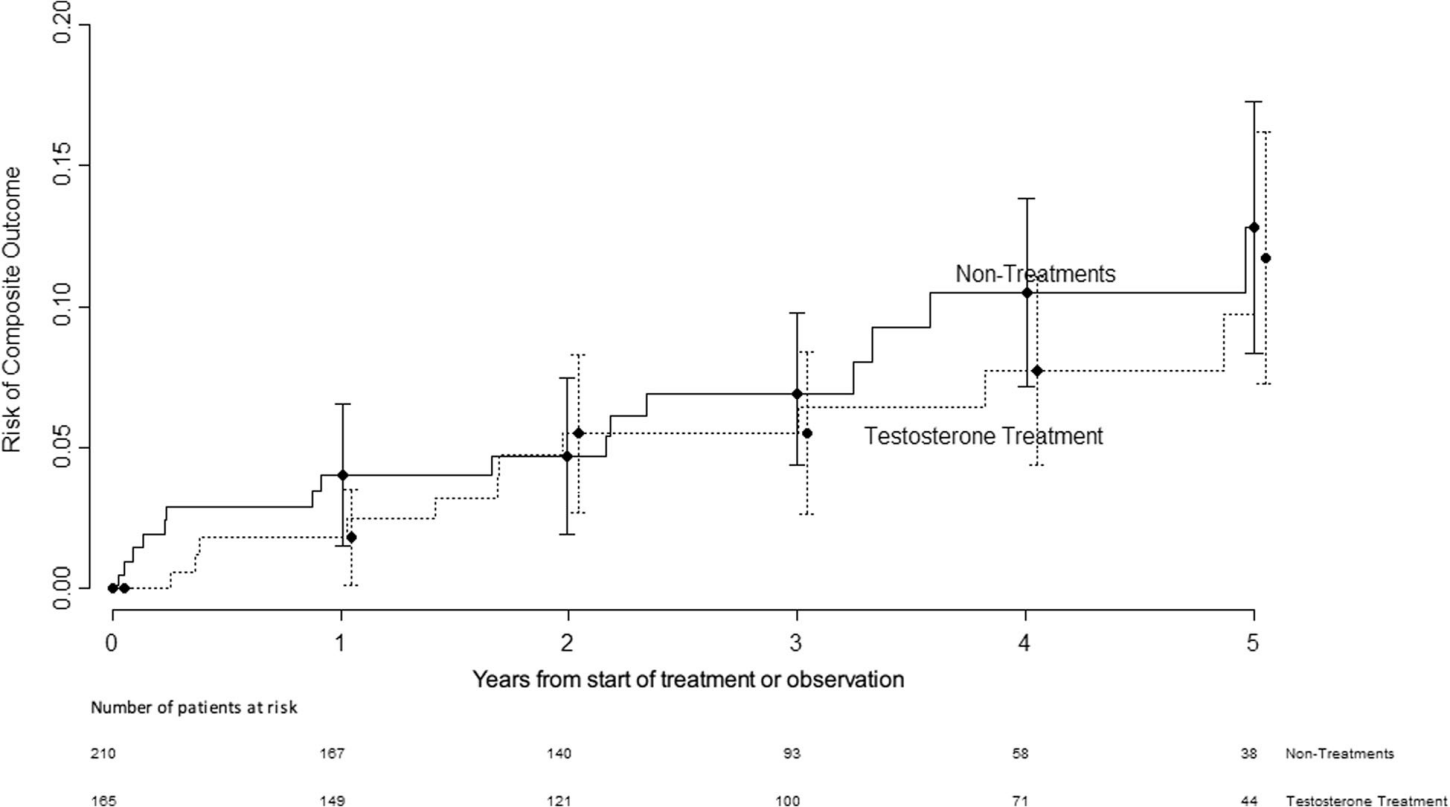
Risk of Composite Outcome over Time with and without Testosterone Replacement Therapy

The median follow-up time was 3.4 (1.9, 5.0) years and 2.7 (1.3, 4.1) years for the treatment and comparison groups, respectively (*P*-value < 0.001). In the treatment group, 12 (7.3%) experienced the composite outcome vs. 16 (7.6%) in the comparison exposed group. The median time (years) to the composite event was 2.1 (IQR 0.9, 4.6) and 1.8 (IQR 0.6, 3.4) for treatment and comparison groups, respectively. No difference in the risk of the combined cardiovascular endpoint was observed between the treatment group vs the comparison group, hazard ratio (HR) 0.81 (95% Confidence Interval [CI]: 0.38–1.71; *P* = 0.5731). Please see Fig. 1.

## Discussion

Our study found that in men with secondary hypogonadism unrelated to overt pituitary disease, with a modest prevalence of CVD, no difference in the risk of the composite outcome in men exposed to TRT vs. those that were not. Our study excluded patients with primary hypogonadism and patients with overt hypothalamic/pituitary pathology because the risk profile of these patients may be different from that of the population of older patients included in our cohort, though many of the studies that have been recently published in the medical literature have included these types of patients [4–7]. A recent study published by Cheetham et al. in 2017 did exclude patients if they had testicular or prostate cancer, pituitary gland disorders, androgen insensitivity syndrome or Klinefelter syndrome, and unlike our report, found that among men with androgen deficiency, dispensed testosterone prescriptions were associated with a lower risk of cardiovascular outcomes over a median follow-up of 3.4 years [8]. It is important to note, however, that the primary composite outcome in this report included more endpoints than did our report, the composite included acute myocardial infarction, coronary revascularization, unstable angina, stroke, transient ischemic attack, and sudden cardiac death. Otherwise, much of the available literature has included many types of hypogonadal patients, identified by low serum testosterone levels, a nonspecific coded diagnosis for androgen deficiency (ICD-9 codes 257.2, 257.8, and 257.9), and/or documentation of testosterone prescriptions, irrespective of the underlying pathophysiology driving the development of hypogonadism.

Low serum testosterone levels have been associated with an increase in cardiovascular (CV) and/or mortality risk in many retrospective epidemiological studies and meta-analyses [16–20]. However, this does not mean that CV and/or mortality risk will necessarily be reduced by treating the low serum testosterone level with TRT. Perhaps the duration of the hypogonadal state, comorbidity burden, etiology of the low serum testosterone, and patient age at the time of initiation of TRT may be more significant drivers of CV/mortality risk than the TRT. Whether low serum testosterone levels are just an association with CV risk and/or mortality risk, or there exists an actual cause and effect relationship, has yet to be answered. Alternatively, low serum testosterone may simply be a marker of overall poor health, and replacing the testosterone may or may not translate into advantageous outcomes. Currently, the only clear indication to provide TRT supplementation to older males with low serum testosterone is largely to improve the symptoms of androgen deficiency, so long as the benefits are felt to outweigh the potential risks [21, 22]. While we excluded patients that would be likely to have contraindications to testosterone treatment, it is certainly possible that TRT may have been avoided in patients perceived by clinicians to be sicker, for factors not included in our matching, and our analysis with matching still may not be properly recognizing these “sicker” patients. Certainly, there is an inherent selection bias that cannot be overcome with a retrospective data analysis, and it was not possible for us to determine why a patient with hypogonadism included in our report may or may not have received a prescription for TRT by their treating physician. Our patients represent a convenience sample of retrospective data and the possibility of selection bias remains.

The 2018 Endocrine Society Guidelines advises against starting TRT in certain populations of men, including uncontrolled heart failure, MI or CVA within the last 6 months [21]. In our report, we could not identify and exclude patients that had an MI or CVA within the past 6 months because identifying the exact date of prior document CV events was not possible. We also did not exclude patients with a history of heart failure. It is difficult to identify the exact dates of CV events with EHR data (particularly events that happened prior to entering our health system) or capture the severity of heart failure (controlled vs. uncontrolled) from the structured EHR data. This information is usually embedded in the free-text progress notes, and not easily systematically extracted from the EHR. In addition, the definition of adverse CV events (MI or CVA) used in this analysis was limited to ICD code documentation in an encounter diagnosis. It is certainly possible that some of the ICD codes recorded were not true CV events. An ICD code may have been entered by a provider with the primary intent of “ruling-out a diagnosis”. The CV events were not validated by manual chart review. These are recognized limitations of our report.

The lower limit that should be used to define a low serum testosterone level is one that is highly debated. The Endocrine Society Guidelines recommend using 264 ng/dL as the lower limit of normal reference range [21], but it should be recognized that the lower limit of the normal reference range is going to vary from assay to assay. We used 220 ng/dL for the lower limit of normal in our report because this was the lower limit of our assay’s reference range during the vast majority of time the patients included in our report were managed at our institution.

Exposure to TRT was defined as having a prescription for TRT documented within the EHR medication list and was not based on pharmacy data. Accordingly, compliance with the prescribed TRT, duration of therapy, and the cumulative exposure are unknown. Furthermore, we did not assess follow-up testosterone levels as a marker of therapeutic exposure, largely because of the heterogeneity of the formulations of TRT that were prescribed, each which has different therapeutic target testosterone ranges. Accordingly, one limitation of these types of health record analyses is the lack of information regarding medication adherence, especially when it comes to self-administered preparations such as topical testosterone products. Our study is also limited in that it only included the structured data contained within the EHR. Information contained in the free-text progress notes was not utilized, however future studies using natural language processing to better define our patient population and treatment modalities would be of great value. Lastly, we acknowledge that our findings could be the result of type 2 error owing to the small number of composite outcomes. The estimated power is about 0.4 when type 1 error is 0.05 and the HR is 0.8.

While our study had numerous limitations, it had many strengths. Patients were matched on what were perceived to be the most important clinical parameters that providers would take into consideration when deciding whether or not to prescribe TRT. Also, our analysis matched patients by their Charlson Comorbidity Score. Accounting for the patients’ comorbidity burden would seem important because the effect of chronic illness may be a confounder impacting the risk of adverse CV outcomes and thus needs to be considered and included in these types of analyses. Matching helped to reduce the bias of potentially confounding variables. SMD is often used as a balance measure of individual covariates after matching. An imbalance is defined as an SMD absolute value greater than 0.10. While there were some slight imbalances between our two groups after matching, per the SMD values, these differences did not appear to be clinically meaningful. In addition, we excluded patients with primary hypogonadism, secondary hypogonadism related to overt hypothalamic/pituitary disease, and the standard contraindications to TRT. Thus, we appear to have isolated the population of patients in whom there is the most concern regarding TRT and CV risk (secondary hypogonadism related to chronic disease, obesity, etc.).

## Conclusions

It is clear that appropriately powered cardiovascular outcomes trials (CVOTs) evaluating the safety of TRT are required to help guide the decision making process regarding whether or not to prescribe TRT to hypogonadal patients. Until the results of appropriately powered CVOTs are made available, our report and the mixed results available within the medical literature, are all that clinicians have to guide their decision making process. In conclusion, our study adds to the growing body of evidence which has suggested TRT offers neither a protective or adverse effect on the risk of MI, CVA or all-cause mortality.

## Abbreviations

BMI: Body mass index
CI: Confidence interval
CV: Cardiovascular
CVA: Stroke/Cerebrovascular Accident
CVD: Cardiovascular disease
EHR: Electronic health record
HCT: Hematocrit
HIV: Human immunodeficiency virus
HR: Hazard Ratio
ICD-9: International classification of diseases, ninth revision
IQR: Interquartile range
LDL: Low-density lipoproteins
MI: Myocardial infarction
SMD: Standardized mean difference
TRT: Testosterone replacement therapy
